# Acceptability and accessibility of child nutrition interventions: fathers’ perspectives from survey and interview studies

**DOI:** 10.1186/s12966-018-0702-4

**Published:** 2018-07-11

**Authors:** Elena Jansen, Holly Harris, Lynne Daniels, Karen Thorpe, Tony Rossi

**Affiliations:** 1 Centre for Children’s Health Research, 62 Graham Street (Level 6), South Brisbane, QLD 4101 Australia; 2 School of Exercise and Nutrition Sciences, Queensland University of Technology, Kelvin Grove, Brisbane, Australia; 3 Institute for Social Science Research, University of Queensland, 80 Meiers Rd, Indooroopilly, QLD 4068 Australia; 4 School of Counselling and Psychology, Queensland University of Technology, Kelvin Grove, Brisbane, Australia; 5 School of Science and Health, Western Sydney University, Sydney, Australia

**Keywords:** Fathers, Feeding, Nutrition, Intervention, Preferences

## Abstract

**Background:**

Against a background of changing family structures and socioeconomic demands in contemporary families, fathers are more actively engaged in meal preparation and feeding of their children, yet in research studies targeting improvement in nutrition and feeding practices fathers are under-represented. Among possible explanations for this bias are *acceptability* of research projects and *accessibility* to male research participants. The aims of this study were to identify (i) fathers’ preferences for participation in child nutrition research and interventions and (ii) the potential to recruit fathers through their workplaces with the possibility of delivering interventions through those workplaces.

**Methods:**

This paper draws on two independent yet linked studies that explored fathers’ roles in family feeding, and intervention studies aimed at supporting father’s dietary knowledge and feeding practices. For Study 1 (conducted first) secondary data analysis was conducted on survey data (*n* = 463 fathers of preschool children) to determine preferences related to type of program, delivery mode, and location and timing. For Study 2 six focus groups and one individual interview were conducted with *n* = 28 fathers to determine acceptability of recruitment of fathers working in traditionally blue-collar occupations and service industries (as defined by the Australian Bureau of Statistics) and potential of intervention delivery through their workplaces.

**Results:**

Fathers were engaged in child feeding and indeed sought nutrition-related information. Fathers indicated a preference for family-focused and online delivery of interventions. Whilst potential to recruit through blue-collar workplaces was evident, participants were divided in their views about the acceptability of interventions conducted through the workplace. There was a sense of support for the logic of such interventions but the focus group participants in this study showed only modest enthusiasm for the idea.

**Conclusions:**

With limited support for the workplace as an intervention setting, further systematic exploration of technology-based intervention design and engagement is warranted. Based on findings, interventions should target a) content that is focused on the family and how to make changes at the family level, rather than the father individually; and b) online delivery, such as Apps or online video chat sessions, for convenience and to facilitate sharing of information with family members.

**Electronic supplementary material:**

The online version of this article (10.1186/s12966-018-0702-4) contains supplementary material, which is available to authorized users.

## Background

Parental feeding practices are potentially modifiable determinants of children’s eating behaviours, nutrition and obesity risk [1–3]. Yet, research examining feeding practices has traditionally focused on mothers as the nutrition gatekeeper within families. This limitation fails to represent the more contemporary division of feeding responsibilities in families where fathers increasingly take a role. Such an approach also excludes family structures that are increasingly common, including single father, same-sex and shared custody arrangements [4]. Domestic roles of men and women have evolved in the last three decades such that mothers work more outside the home most often in paid work [5] and fathers now spend more time in household responsibilities and child rearing [6]. Recent advances in father-inclusive research have shown that fathers are involved in child feeding responsibilities [5] and can have an influential effect on their child’s eating, physical activity and weight [7–11].

Recruitment of fathers in child obesity research however has been challenging [12]. When over 300 fathers in the USA were asked why they believed fathers participate less than mothers in child health research, 80% said it was because they were simply not asked [13]. Of observational studies examining links between parenting and childhood obesity published between 2009 to 2015 only 10% have reported results on fathers [14]. A recent systematic review indicated that only 6% of all parent participants in childhood obesity treatment and prevention interventions (between 2004 to 2014) were fathers [15]. Overall, fathers are substantially under-represented in child nutrition research and interventions that aim to understand and modify determinants of childhood obesity risk [16, 17]. Whether fathers are interested in participating in child nutrition interventions is unclear. Importantly, whether the design of research (including intervention aim and content, mode of delivery and location) act as barriers or enablers of participation is not understood. Harnessing fathers’ participation in child obesity prevention requires an understanding of how to effectively access, recruit and engage fathers in child nutrition research and interventions.

Framing of research is key for including fathers in child health research, specifically the language used for recruitment purposes and the structural elements of interventions [12]. The current study focuses on preferences for nutrition interventions within the family context. Studies targeting both parents typically recruit mothers, who are then asked to approach their partners [18]. This enables mothers to opt fathers in or out of research. While seemingly cost effective, accessing fathers via mothers may homogenise samples, for example, participating fathers are likely to have a higher education/income, be married or be in a positive relationship with mothers [19, 20]. Recruitment via father-focused venues, such as workplaces, may circumvent biased samples [13]. In Australia, 92% of fathers of children aged 4- to 5-years old work full-time (compared to 46% of mothers), [21] which in itself, could be a barrier to accessing fathers. Little is known about the efficacy of recruiting fathers in the workplace for research in the areas of obesity, family feeding or nutrition interventions. In research focused on men’s weight loss, an intervention in the context of a male-dominated workplace has been successful in reducing men’s weight and improving indicators of dietary intake [22]. Engagement through the workplace to specifically target fathers in supporting their child’s health and eating behaviours has not been explored.

The purpose of this research was to assess fathers’ interest in participating in child nutrition interventions and identify their preferred intervention focus, mode of delivery, and gauge their opinion regarding the viability of their workplace as an intervention location. Data for this paper are drawn from two independent but linked Australian studies specifically focused on the inclusion of fathers, one a quantitative cohort survey and the other a qualitative focus group interview study. Sequentially the research was organised first, to look at fathers’ participation in interventions via a cohort study which was then followed by a qualitative study to more closely investigate the underlying processes associated with participation.

## Study 1: Cohorts analysis

### Methods

#### Study participants and procedures

Study 1 analysed data from the ‘Father’s Feeding Participation and Practices’ study (FFPP) [23, 24], conducted in 2011. The FFPP aimed to examine the nature, extent and predictors of involvement and the influence of fathers in feeding preschool-aged children as a key determinant of child health. The study collected new data from fathers with healthy preschool-aged children (2- to 5-years old) via two pre-existing cohorts and a new sample. Two community-based family samples were utilised to recruit fathers for FFPP via participating mothers: 1) NOURISH, a randomised controlled trial of Australian first-time mothers from primarily middle-class parents [25] and 2) Environments for Healthy Living (EFHL), a birth cohort study in low income families [26]. An additional sample, initiated by FFPP, recruited university staff and students via their faculty email distribution list using convenience sampling [23, 24]. All recruited participants were asked to complete a questionnaire via hardcopy or online formats, about their (oldest) 2- to 5-year old child. They were not provided with any incentive for participation. The return or online submission of the completed questionnaire was accepted as an indication of fathers’ consent to participate. While the overall response rate could not be calculated, the response rate from the NOURISH and EFHL sub-samples was approximately 20% each [23]. In total, 436 fathers from diverse socioeconomic backgrounds provided valid responses. FFPP was conducted in accordance with ethical approval from Queensland University of Technology and Griffith University Human Research Ethics Committees.

#### Measures

Sociodemographic characteristics were self-reported by fathers including age, level of education and hours of paid work per week. Fathers also reported their weight and height, relationship with the child for whom they filled out the survey, the number of days in an ‘average’ fortnight that they lived with their child, relationship status, child age and gender.

Fathers’ confidence and knowledge of healthy eating (6 items, e.g. “I am confident that I can prepare healthy food for my child”, α = 0.81), perceived responsibility (5 items, e.g. “How often are you responsible for deciding what your child eats”, α = 0.94) and attitudes about paternal involvement in child feeding (2 separate items, e.g. “I would like to be more involved in feeding my child”) were assessed with validated [27] and study-specific items (see Additional file 1). Response options for all items were on a 5-point Likert Scale (1 = strongly disagree/rarely to 5 = strongly agree/mostly). The mean score was calculated for confidence/knowledge and perceived responsibility, with higher scores indicating more endorsement.

To assess fathers’ interest in and preferences for participation in nutrition interventions, several separate study-specific questions were asked. These questions and response options were conceived by expert consultation, including two of the authors with specialised knowledge in family feeding interventions (KT and LD). All responses were measured on 5-point Likert Scales (1 = strongly disagree/very unlikely/not at all to 5 = strongly agree/very likely/very). Interest in learning about nutrition, which is a possible pre-requisite for participation in any intervention, was measured with two items (“I am interested in learning more about healthy eating for myself” and “I am interested in learning more about healthy eating for my child”). Fathers were then asked to indicate how likely they were to participate in the following types of healthy eating program (i.e. intervention focus): individual, group, family, and fathers only program. Ratings of usefulness of delivery modes included: online, interactive social network (e.g. online group forum), DVD (e.g. information DVD), written (e.g. information booklet), and mobile phone (e.g. SMS or text message). Finally, fathers were asked to rate their preference for the location (i.e. in your community) and timing of a nutrition intervention (i.e. after work hours or on weekends). Descriptive statistics were conducted in IBM SPSS Statistics version 23.

## Results

Characteristics of the fathers participating in Study 1 are shown in Table 1. Self-rated knowledge and confidence of fathers relating to child feeding was very high (M = 4.35, SD ± 0.51). The majority of fathers (72%) agreed (moderately/strongly) that they *should* play an equal role to mothers in feeding their children. In contrast, current responsibility for making decisions relating to what and how much their children eat was low (M = 2.71, SD ± 1.02). Twenty-four percent of fathers indicated (moderately/strongly) that they would like to be more involved in feeding their child. Fathers were highly interested in learning more about healthy eating, but expressed a preference for a program relating to their child’s eating compared to their own (with 67% compared to 51% moderately/strongly agreeing). This provides an important picture of fathers’ engagement with healthy lifestyle programs and for participant recruitment strategies. Our results reveal that when child health and wellbeing are the *focus* of intervention strategies, fathers in this overall research program at least showed some enthusiasm for engagement.

**Table 1:** Characteristics of fathers participating in two child feeding studies and their children

Variable	Father’s Feeding Participation and Practices Study (*N* = 436)	What Fathers Want Study (*N* = 28)
	Mean ± SD or %	
*Father*		
Age (years)	37 ± 6	41 ± 6
Biological father	98	93
Live with child every day during ‘average’ fortnight	89	82
Married/defacto	97	86
Level of education		
No university degree	66	64
University degree	34	36
BMI^a^ (kg/m^2^)	26.9 ± 4.1	–
Healthy weight (BMI < 25)	38	
Overweight (BMI ≥ 25 and < 30)	46	
Obese (BMI ≥ 30)	16	
Hours of paid work/week	41 ± 15	40 ± 7
Less than full-time (0–35 h/week)	16	4
Full-time (≥ 35 h/week)	84	96
*Child*		
Age (years)	3.5 ± 0.9	–
Gender (boy)	53	–

^a^BMI was calculated based on self-reported weight and height

*BMI* Body Mass Index, *Dash* data missing as questions were not asked in the What Fathers Want study

In terms of fathers’ willingness to participate in different types of healthy eating interventions, a family-focused program appealed to most fathers (58% were somewhat or very likely to participate), while fewer would consider an individual (32%), group (23%) or fathers-only (23%) program. Regarding fathers’ rating of the mode of healthy eating information delivery, fathers identified online programs as the most popular option (70% said this mode was moderately or very useful); followed by written information (62%), DVD (56%), interactive social networks (30%) and mobile phones (17%). The presented options for location and timing of healthy eating programs appeared to be of only modest appeal to fathers. Based on the findings that 46 and 42% of fathers respectively indicated that they were unlikely to participate in a healthy eating intervention conducted in the community or after work/on weekends, a subsequent qualitative study was designed to investigate if workplaces were preferable. The researchers theorised that this might be a viable option for men in work, given the time that is usually devoted to work. The recruitment decisions were guided by the Australian Bureau of Statistics recent descriptions of occupations. Of particular interest were men in occupations characterised as skilled, semi-skilled or low skilled manual labour (“blue collar”). These types of workers are known to be harder to reach (see [28]), more often work unsociable shift hours, and are on average less well educated than those in professional occupations [29]. The participants included heavy good drivers (requiring specific licensing) and other delivery workers, postal/mail room workers, warehousemen, and food service personnel.

## Study 2: Focus groups

### Methods

#### Study participants and procedures

Study 2 analysed data from focus groups conducted in the ‘What Fathers Want’ Study. The overall aims of the ‘What Fathers Want’ Study were to (i) determine fathers participation in their children’s mealtimes, (ii) determine the accessibility and feasibility of recruiting and conducting research related to children’s health and wellbeing with fathers through their workplace, and (iii) explore fathers’ perceptions of workplaces as a potential setting to implement family-focused nutrition interventions. In this paper we primarily report on fathers’ perceptions regarding the intervention delivery through their work (aim iii; i.e. acceptability), but also briefly discuss the feasibility for recruitment through workplaces (aim ii; i.e. accessibility). The aim was to recruit fathers from workplaces with ‘blue collar occupations’ and ‘service industries’ as defined by the most recent Australian Bureau of Statistics (ABS) [30]. These include technicians and trades workers, machinery operators, drivers, and labourers as well as occupations within health care and social assistance, postal and warehousing, and accommodation and food services [30].

**Fig. 1:**
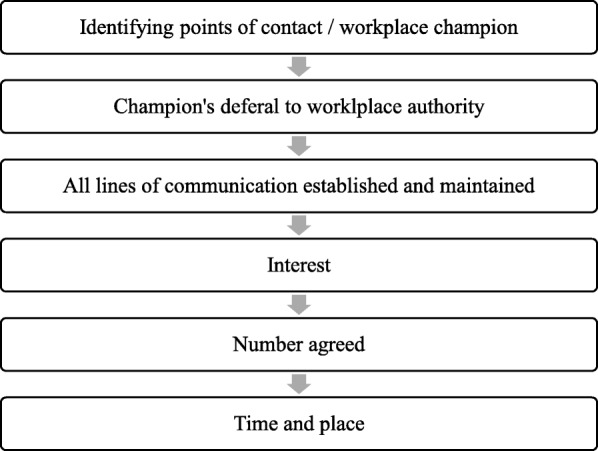
Flowchart reflecting intensity level of recruitment – overivew of actions taken within each recruited workplace

A three-pronged recruitment strategy was implemented to access a diverse range of fathers and their workplaces. Initially, workplaces were identified from the Queensland Government website ‘Healthier. Happier. Workplaces’ [31]. These workplaces are recognised for developing strategies and policies to implement best practice workplace wellness programs that aim to improve the health and wellbeing of employees. Those listed on the recognition wall for achieving bronze, silver or gold recognition and located within South East Queensland were approached. Secondly, the research team attended a men’s health expo held in a city in Queensland to connect with the community and possible workplaces. Finally, the research team used established networks within local industry groups to individually contact fathers and gauge interest at their workplaces. Important in this strategy was the identification of workplace health or wellness ‘champions’ if these existed, to assist with the recruitment process. Not all workplaces have such a person. The identified workplaces were then emailed (*n* = 27) and called to follow up (*n* = 19) after which the research team followed the steps indicated in Fig. 1. Field notes indicated that the main reasons for workplaces declining to participate included: failing to respond via email or telephone, workplaces advised that there were no fathers with children in the 0- to 12-year age group, busy shift schedules and inability to get a group together off work-related tasks at the same time, or they were uninterested in the study.

Qualitative methodology was optimal given that a key aim of the study was to elicit fathers’ views of the workplace as a viable site for delivery of family-focused nutrition interventions. Of equal importance were fathers’ accounts of their role in formal family mealtimes or other feeding requirements/opportunities within the family structure. It was recognised that there would not be one identifiable objectionable truth, but rather multiple truths or at least realities that have been constructed through the lived experience. Eisner [32] comments that “meanings are construed, and the shape they take is due, in part, to the tools people know how to use” (p. 36). Therefore, it is reasonable to suggest that fathers ascribe multiple meanings associated with recruitment through workplaces, views associated with the workplace as a point of intervention, feeding practices within households and fathers’ roles within these. Due consideration was given to the type of interview structure that might yield data that might be of most interest. Focus group conversations have been widely (albeit relatively recently) used in health research [33] and extensively for a longer period in social research [34]. Primarily, the choice of focus groups was guided by Guest and colleagues [35] who found in a randomised study that men in focus groups were more likely to reveal personal and even sensitive material than they are in individual interviews [36]. Additional advantages of focus groups include: generating data through interactions between participants, within the context of the workplace (setting was important for data collection); [37] distinguishing shared and non-shared experiences within the group, developing an understanding of the range of perspectives and appearances; [33] and enabling participants to query and explain processes/ perspectives to each other [33]. Focus groups are also considered to work well with relatively homogenous groups where homogeneity might be defined by one or more characteristics (such as occupations/jobs and gender) [38]. Whilst not a prime reason, it is worth noting that a focus group approach offered some logistical efficiencies in that it enabled the research team to *meet* with more participants relative to the allocated trips to the field where workers would be meeting immediately before or after work (or shift), or in breaks during the working day (or shift). This study was approved by the human research ethics committee of the Queensland University of Technology.

Focus groups were organised on site and during working hours or immediately before or after. Respondents at six different workplaces agreed to participate. In total, 28 fathers agreed to participate and were interviewed across six focus groups (3–7 participants) and one individual interview. The final number at each worksite was dependent on the time and resources of the participants. The focus group conversations were conducted by three members of the research team (EJ, HH and TR). The earlier sessions were led by an experienced qualitative researcher (a male) with extensive experience in interviewing, focus groups and broader qualitative fieldwork in the areas of education, sport and workplace learning in a range of community and institutional settings (TR). Progressively other female team members took the lead at different locations (HH, EJ). At the beginning of the focus groups or interview, participants were asked to complete a short demographic survey. Characteristics included father’s age, level of education, relationship with child, the number of days in an ‘average’ fortnight that they lived with their child, relationship status, level of education, and hours of paid work per week. Indicative questions are shown in Table 2. These were largely based on the literature but also on findings of Study 1 that left much detail about fathers engagement in feeding and their desire/interest for further support in the form of interventions untouched and unsaid.

**Table 2:** Indicative questions for focus group discussion – Study 2

Indicative question	
1. Think about meals you have eaten with your child/ren, within the last 2–4 weeks. Can you tell me what happens at mealtimes with your youngsters?	
2. When it comes to feeding the family, what are your jobs?	
3. What child feeding jobs do you share with another adult in the household?	
4. What do you like about mealtimes with your family?	
5. Sometimes feeding the kids can be challenging. What challenges or worries have you experienced with your kid/s at mealtime?	
6. Can you tell us what happens at mealtimes when your child doesn’t like the food being served?	
7. If you were to encounter challenges with your children around feeding or nutrition, would the workplace be a viable place to receive more information or advice?	
8. Tentative question: what would you like to change when it comes to family meals?	

#### Analysis

To analyse the data an inductive approach was taken, guided by the principles of grounded theory. Inductive approaches “primarily use detailed readings of raw data to derive concepts, themes, or a model through interpretations made from the raw data by an evaluator or researcher” [39]. The research team members independently conducted a structured hierarchical analysis of the transcribed interview data of one of the focus group discussions (the very first one undertaken). All researchers therefore reviewed the same document privately. Hence, each researcher independently read, and re-read the transcript, applying labels, tags and codes to create ‘meaning units’ (MU) as a first analytical level to create a broad narrative from the focus group conversation (see Côté et al. [40]). A meaning unit has been described by Tesch [41] as a segment or section of text that contains a single idea, an episode or mini event, or a snippet or information such that the MU can stand on its own out of the context of the textual data. The researchers then collaboratively reviewed the meaning units across a further analysis session of three hours. This was to establish agreement in observations, tags and codes that formed the meaning units and to eliminate what appeared to be extraneous codes and identifiers. The MUs were then clustered to create categories. Once the categories had been refined in this way and agreed upon, each researcher was then allocated an equal share of the remaining transcripts to analyse. Upon completion of this phase the researchers gathered again to create larger overarching categories that were then referred to as themes and sub-themes creating a hierarchical structure similar to that described by Côté et al. [40]. This then showed the overarching themes in the data, sub-themes that sat beneath each overarching theme, and the contributing categories with key identifiers of each category. Under each of the key identifiers the textual data from the transcripts could then be listed. This process is also consistent with the guidelines for grounded theory analysis as established by Strauss and Corbin [42] and was intended to reach the point of thematic saturation a common goal in qualitative research [43]. It is acknowledged that ‘saturation’ can be a contentious term. In this case the focus was on what Hennink and colleagues [43] call ‘meaning saturation’. That is, the data were analysed for themes and structures such that a point was reached where new themes and structures could no longer be built from the meaning units or categories. The analysis enabled an ascending or descending reading of the hierarchical structure of the data. Based on the coding and collaborative review processes, a research narrative was constructed with indicative participant text as exemplars.

## Results

### Participants

Study 2 achieved access to and recruitment of fathers through their workplace. The workplaces or workplace departments from which the participants were drawn have traditionally been considered and indeed categorised as ‘blue collar occupations’ and ‘service industries’. Within contemporary work environments, this description fails to capture the multiple profiles of workers within a single organisation. It is more accurate perhaps to identify the workplaces from which the participants were drawn to clarify their occupational identity. The workplaces within which the focus groups took place included: transportation services, construction and maintenance services, postal/mail room services, social assistance and food service personnel. Examples of self-described work included mail delivery, driver and transport dispatch manager. Further demographic characteristics of fathers are presented in Table 1. In the following, abbreviations such as “FG4, M1” are used to highlight the focus group and member who provided the specific textual data.

### Preference for the workplace as a setting for nutrition interventions

The data analysis enabled construction of two broad themes, including (a) advice (type and nature of advice either taken or sought) and (b) intervention (preferences for and commitment to types of intervention within the workplace). The ‘intervention’ theme provided the focus with particular reference to the workplace as a site of intervention. In addition, elements of the ‘advice’ theme were included specifically where it applied to the idea of interventionist strategies. We present these two themes in the form of a single narrative for the purposes of flow, which is more representative of how these data emerged in conversation.

During the six focus groups and individual interview, fathers discussed their views on using their workplace as point of contact for interventions. Overall, fathers were only mildly attracted to the idea of having a family-focused intervention in their workplace.

*“Interviewer: Yeah, like covering some topics like family nutrition, is that something you think you guys would be interested in?”*

*“Father: Not really. I mean I wouldn’t. I don’t think I would. Certainly not that I don’t value that, but I wouldn’t.” (FG4, M1)*

*“It might help some people. I don’t reckon it would help me. I’m just not that way inclined…” (FG1, M2)*

Hence a rather paradoxical view was presented, while the fathers acknowledged the logic of nutrition interventions being conducted at the workplace, their own enthusiasm for participation was mixed showing only modest enthusiasm for the idea. They agreed that the workplace would be a pragmatic setting, because of the long hours they spend there. Fathers recognised that leaving work to attend an appointment or intervention outside was problematic.

“*There would be a much better chance of me doing something in the workplace rather than in private time.” (FG1, M1)*

*“It’s definitely more convenient, 100% more convenient. So when we are all doing probably 50-55 hours a week, and then there is travel time to and from home. During the week the kids play sport, sports training and all sorts of other stuff. There is very little time to do much else so anything you can do in the workplace, definitely. You are more likely to take advantage of it definitely.” (FG1, M2)*

*“You’re there for 8 hours a day, so where else are you going to get it.” (FG6,M1)*

However, the fathers in this study were reluctant to commit to such interventions in the workplace. In doing so they expressed several barriers, which prevented their particular commitment to attend such intervention programs if they were available through their workplace. Fathers indicated the plethora of information/advice regarding child nutrition that is available beyond the workplace, if they were inclined to access such information. The role of a dietitian or health professional specialising in nutrition was acknowledged only once as a credible source of information across the entirety of the focus group conversations. Other sources of information or advice were certainly more convenient but little was said about the credibility of the available information.

*“I think there are lots of information already out there if you are willing to look. […]” (FG7, M3)*

“*For general issues. But for more specific individualised nutritional challenges I’m sure the Accredited Practising Dietitian might be a better starting point.” (FG7, M4)*

Furthermore, fathers raised issues of agency. More specifically, fathers expressed their own initiative in seeking child nutrition advice, be it through self-initiated behavioural interventions based on advice from their partner, media coverage and alternative medicine, or even a simple visit to the pharmacy.

*“When she reached 9 months, two years or three years old, she just eat the half of bread or when we’re eating rice, she eats only a couple of spoons and then sit. […] I took her to a family doctor, and then when she reach four or five years old she starts to eat well. Now she is seven years old and she’s still the same. […] I went to pharmacy and we bought vitamins, it works just within two to four days.” (FG2, M3)*

*“Preservatives and numbers and the sugar and I guess the effect that has on kids - that would be what I would be interested in, yeah. My friend is a naturopath and she’s given me things to read about, you know, that sort of stuff.” (FG4, M5)*

Similar to fathers in Study 1, those in Study 2 reported confidence in their knowledge about nutrition. Interestingly, there was a perception of some respondents that a nutrition intervention would resemble top-down control and ‘policing’ of food intake.

*“Yeah, like, I wouldn’t say that I know everything. I think that workplace education would be handy to come from a point of knowing your body and knowing yourself and knowing what you can do. Not based around – ‘you must eat this, because it has got this calorie count’ or ‘this is low fat, you have to eat that’. That’s a load of crap.” (FG2, M4)*

Notably, other sources of information, such as friends and documentaries, were deemed easy to access outside work largely because they were not time nor in most cases media constrained. Credible sources were identified as is seen in the following text examples. This though was also interspersed with more questionable sources (friends, the internet):

*“Probably because the other things are more readily accessible. If you’re at home you can just ring the health line and call a friend or something like that. I haven’t really been in a situation yet where*. *.*.” *(FG3, M1)*

*“It normally happens when you’re at home. The most regularly one is either the Internet or the doctor. You call them to ask them. So far it hasn’t happened when I’m at work.” (FG3, M2)*

These textual data show a likelihood that this intervention might only be warranted to solve a particular problem or difficulty rather than for educational or preventative purposes. While it is not possible to claim generalisability to other fathers or to other workplaces here, this perspective perhaps warrants further investigation since seeking advice only at a point of potential difficulty could be considered as concerning. From a health promotion or prevention perspective this behaviour is considered reactive or ‘too late’, while the sources of support that are mentioned here are considered less than optimal.

Some fathers argued for the possibility of nutrition interventions to be integrated into (rather than additional to) the workplace health and safety program, albeit again with tepid endorsement. More enthusiasm was shared for integrated wellness programs that supported fathers’ own individual health, a contrast with reports from Study 1.

*“I think that you can take personal lessons and then deliver them back into the home environment. It’s the same in terms of the emphasis that we make in our safety training. You can contextualise it back to someone’s personal life, and often that is actually the thing that ticks through as opposed to, ‘this is the work environment,’ and they can take that safety culture back home. […]We’re actually seeing a greater emphasis now on people’s health and through education in people’s health and connecting that into the workplace as well.” (FG2, M2)*

*“We also last year where we did a bit of a drive and we got all the drivers in for a 30 minute health check with health professionals. […] People really appreciated being given the opportunity during their work hours to come and do it and take something away from it.” (F1, M2)*

Nevertheless, some fathers acknowledged the suitability of nutrition information delivered in the workplace setting via a face-to-face setting, or an optional extra which can be accessed through newsletters or within-organisation electronic communication platforms.

*“I don’t know how the project is. But I reckon that if first of all if you are creating fliers, papers, things that it could suggest in general in terms of child food or something like that and where we could access through the [company] could be very handy.[…] I recommend a weekly paper talking about children’s food or something like that, children’s health, weight—things that are important for their health” (FG3, M2)*

*“It would be more readily available. Like the one on the computer when someone’s having a problem someone can just access it quickly and through the internet.” (FG3, M4)*

## Discussion

Fathers influence their child’s feeding and nutrition [10, 44]. However, fathers remain under-represented in research on family mealtime participation and child feeding practices [15]. A key aim of this paper was to examine fathers’ accessibility for and acceptance of participation in child nutrition interventions and research. We presented two independent but conceptually linked studies, the first documenting the extent of fathers’ interest in different types and modes of nutrition and feeding intervention delivery and the second their underlying intent and reasoning for (lack of) participation at their workplace. Through both survey and interview methods, we found that fathers are involved in child feeding. However, they raised issues of acceptability of nutrition interventions, including variable levels of interest in participation depending on the aim, content and location of delivery. Since acceptability of child nutrition interventions is necessary but not sufficient for successful implementation, accessibility to fathers for child nutrition research was also examined. In Study 1, we obtained a response rate of approximately 20% from fathers whose partners were already involved in cohort studies, inevitably introducing selection bias. To expand our understanding of fathers’ preferences for intervention from ‘hard to reach’ populations, we specifically attempted to access fathers in Study 2 who were ‘skilled, semi-skilled or low skilled manual workers’ (“blue collar”). Our work highlights the importance of first evaluating fathers’ preferences for interventions before developing and delivering culturally appropriate interventions.

Despite fathers’ reported interest in participating in family-focused nutrition interventions, they reported low commitment to participate in the community or outside of work hours (Study 1). Yet, when fathers were asked if they believed the workplace is a potential setting for the delivery of family-focused nutrition interventions, the responses were only moderately enthusiastic (Study 2). These findings underscore the significant challenge in involving fathers in nutrition interventions even when they described high engagement with and interest in feeding their children. Our data from both Studies 1 and 2 indicate that although fathers express interest in their children’s health, the workplace as a site of advice, education or intervention was not favoured.

Fathers showed interest in learning more about nutrition, particularly for their children or the whole family. Consistent with previous research [14], including that conducted in urban fathers with limited income [45], interventions that enable or strengthen the capacity of fathers in supporting their family, rather than being targeted to their own health, are recommended. Our finding also support research by Morgan and colleagues [9, 22], which demonstrated the successful recruitment of fathers and their children into the “Healthy Dads, Healthy Kids” program. Our work shows that fathers are interested, and perhaps more willing to participate, in interventions when their children are the focus of the program. This might offer new possibilities for lifestyle program recruitment. Furthermore, fathers believed that they were knowledgeable and confident in feeding their children. Whether this confidence aligns with father’s nutrition knowledge was not tested in our study. Despite current reported levels of knowledge and confidence, fathers in both Studies 1 and 2 expressed how they continue to seek additional knowledge. High confidence, independent of knowledge level, may prevent some fathers from participating in some types of child nutrition interventions and also raises a key question about critical evaluation of knowledge sources. Our data from Study 2 indicate that fathers’ self-directed search for nutrition knowledge was directed to a diversity of sources including print and web-based media, friends, alternative therapists and a diversity of health professionals. They did not discuss concerns related to the veracity of the information nor its underlying evidence base. Our data suggest that such information seeking was often initiated in response to a feeding problem, for example when children’s behaviours or tastes changed. The findings identify potential opportunity for interventions to increase critical analysis, and targeting provision of an evidence-based and enjoyable website for fathers and parents more broadly.

In Study 1 and 2, fathers reported low but increasing levels of feeding responsibility in their home, however they did express a positive attitude to being involved in feeding and the increasing level of obligation as food labour in the home became more distributed. More than two-thirds of fathers in Study 1 preferred online programs (but not online group forums) as a mode of delivery. An online program delivered to mothers of pre-school aged children showed improvement in children’s dietary intake [46]. Similarly to the Growing Healthy study [47] for parents living in socioeconomically disadvantaged areas, there may be benefits in engaging fathers online and future research could evaluate this mode of intervention particularly through online video chat sessions or Apps that can monitor and calculate families’ eating (e.g. dietary intake) and feeding (e.g. frequency of responsiveness to cues) behaviours but also those that can act as alert devices when dietary intake or feeding interactions could be improved. Paramount to this evaluation is teaching fathers how to assess the quality of evidence and information available online. Future interventions also could include developing material for fathers, by fathers, to produce “socioculturally relevant” (p. 8) [15] material. It is important to note that no intentional information was gathered about father’s preference for face-to-face delivery of nutrition interventions in this study though interestingly it emerged as a point of discussion in Study 2. In contrast to previous suggestions [48], fathers expressed little interest in attending a nutrition intervention within their community or outside of work hours. Therefore, the possibility of family-focused nutrition interventions implemented within the workplace was explored in Study 2. ‘Employee assistance programming’ at work has gained popularity, likely due to their potential to enhance productivity of parent employees [49].

In Study 2, fathers had mixed responses to the proposal of the workplace as a setting for the delivery of family-focused nutrition interventions. Fathers acknowledged the potential capacity of the workplace to a host nutrition intervention; an acknowledgement based largely on the time fathers are perceived to spend at work. Fathers also openly discussed reasons for their lack of interest in participation. Trust in the personnel delivering the intervention appeared paramount to fathers. Somewhat unexpectedly there is little or no evidence of the public’s perceptions or ‘trust’ of nutrition or dietetic allied health professionals [50] and more research (particularly with fathers) is warranted to understand who may be the most trusted personnel of such delivery of an intervention. Davison et al.’s [14] findings showed that the reputation, possibly linked to trustworthiness, of the leading organisation of an intervention is critical in whether fathers would participate in child health research. Contrary to Study 1, fathers in Study 2 appeared to be more interested in workplace health interventions targeted at individuals, particularly if these workplaces already offered a service into which nutrition could be folded. Whether these interventions could indirectly positively influence eating behaviours within the whole family, through paternal modelling, [10, 44] is not well known and warrants further investigation.

While, from the fathers’ perspective in Study 2, the workplace may not be the ideal choice for delivery of interventions, findings suggested, in line with Palm and Palkovitz recommendation [51], that workplaces are a potential ‘access point’ to interact with fathers. Recruitment of workplaces for the What Fathers Want Study was labour intensive. The recruitment process required a systematic plan and sustainable human and economic resources (for example, each participating father received an AUD20 gift voucher for a hardware store). Comprehensive and high-intensity recruitment strategies (email, telephone call, face-to-face contact) were required to obtain the focus group sample of fathers. Face-to-face recruitment (e.g. via the Men’s Health Expo or established networks) was a particularly helpful strategy. Forming collaborative relationships with community stakeholders or key contacts (identified as ‘workplace champions’) has previously been reported as beneficial for the recruitment of fathers [13]. Depending on the structure of the workplace, several layers of contact are possibly required until contact is made with the actual study participants. This recruitment process included co-dependence on the ‘workplace champion’ to gain access into the workplace. Once access to the workplace was granted, focus groups were organised via the workplace champion. On the arranged day, the number of fathers participating in the focus group often (and surprisingly) exceeded that expected based on the number of fathers expressing interest to the workplace champion. ‘Peer recruitment’ has previously been described as effective strategy, [52] however, this also highlights the need for flexibility and preparedness (e.g. more vouchers needed than planned). Under the proviso of a high-level of commitment, accessing fathers in the workplace was a suitable method for father-focused recruitment, likely to result in less demographically-biased samples compared to mother-focused recruitment of fathers [19, 20]. In addition to biased sampling, indirect recruitment of fathers through mothers, as done in Study 1, may not be very effective either. Future research projects could recruit fathers through the workplaces, but acknowledge that the workplace may not be the site of intervention and that ability to recruit there would be contingent upon workplace agreement. Workplaces may additionally present an effective platform for gathering information from fathers about intervention design and implementation (e.g. piloting material).

### Strengths and limitations

Taken together, our research provides evidence regarding fathers’ preferences for family-focused nutrition interventions. Recruited fathers in Study 1 were representative of Australian men in terms of education levels and working hours, as national data indicates that 33% of males aged 35–44 years old had completed a university degree [53, 54] and 92% of fathers of children aged 4- to 5-years old work full-time [21]. In contrast, fathers were less representative according to their weight status with national data indicating that 71% of men were overweight or obese [55]. Notably, since weight and height were self-reported in our sample, there is the possibility for underreporting. Fathers self-selected to participate in the studies and may have been more involved in feeding their children. As previously reported [23, 24], fathers of Study 1 showed high levels of engagement in feeding their children, which may reflect selection bias and hence limit generalisability of the findings. Fathers who are currently less involved or interested in feeding their children will likely be even harder to reach, recruit and retain in child nutrition research and interventions. Both male and female researchers interviewed fathers in Study 2, which may have influenced the response of participants. While in the 1990s male discussion leaders were recommended [45, 48], Davison et al. showed in 2016 that 73% of fathers believed that interviewer gender did not matter [14]. The data for Study 1 were collected in 2011 and therefore have not investigated other technologies which have gained popularity since then (e.g. smart phone apps). Unfortunately, neither of the studies specifically asked fathers where and when they preferred attendance for family-focused nutrition intervention and this warrants serious further investigation. Future studies also need to investigate if fathers who are working in other types of industries may or may not prefer family-focused nutrition interventions through their workplace.

## Conclusion

This paper contributes to understanding of both the need and means by which fathers are engaged in family-focused nutrition interventions, and presents a unique approach in converging findings from two methodologically differentiated studies. Data from both studies showed that face-to-face interventions delivered in the workplace or broader community were not favoured. Online options were preferred as highly flexible and accessible modes of delivery of nutrition and feeding knowledge and skills. The findings of the quantitative cohort studies and rich qualitative study converge to identify online as optimal form of intervention but raise the question of appropriate search strategies and the need for a site design that is “problem focussed” as feeding and nutrition problems were identified as a catalyst for web-searching given that the preference seems to be for self-initiated strategies in searching for information, dietary trends, and expertise.

## Additional file

Additional file 1: Overview of constructs assessed in Study 1, including their respective items, response scales and internal reliability. (DOCX 19 kb)

## Abbreviations

ABS: Australian Bureau of Statistics
BMI: Body Mass Index
EFHL: Environments for Healthy Living
FFPP: Father’s Feeding Participation and Practices Study
