# Supplementary material for: Acceptability and accessibility of child nutrition interventions: fathers’ perspectives from survey and interview studies
Source: Int J Behav Nutr Phys Act. 2018 Jul 11;15:67. doi: 10.1186/s12966-018-0702-4 (PMC6042245; doi:10.1186/s12966-018-0702-4)
Supplement: Supplementary file 1 — Overview of constructs assessed in Study 1, including their respective items, response scales and internal reliability. (DOCX 19 kb) [file 12966_2018_702_MOESM1_ESM.docx]

**Additional file 1**

Overview of constructs assessed in Study 1, including their respective items, response scales and internal reliability

| Construct | Items | Response scale & reliability |
| --- | --- | --- |
| Confidence/ knowledge | “I feel I know enough to be able to choose a healthy diet for myself”  “In general I have a healthy diet”  “I know what my child should be eating”  “I know how much my child should be eating”  “I am confident that I am able to feed by child well”  “I am confident that I can prepare healthy food for my child” | 5-point Likert Scale (1 = strongly disagree to 5 = strongly agree)  α= 0.81 |
| Perceived responsibility | “How often are you responsible for organising meals for your child?”  “How often do you decide how much food is offered to your child?”  “How often are you responsible for deciding if your child eats the right kinds of food?”  “How often are you responsible for deciding what your child eats”  “How often are you responsible for deciding how much your child eats?” | 5-point Likert Scale (1 = rarely to 5 = mostly)  α= 0.94 |
| Attitudes | “Fathers should be as heavily involved in the feeding of their children as the mother is”  “I would like to be more involved in feeding my child” | 5-point Likert Scale (1 = strongly disagree to 5 = strongly agree)  Separately used |
| Interest in learning about nutrition | “I am interested in learning more about healthy eating for myself”  “I am interested in learning more about healthy eating for my child” | 5-point Likert Scale (1 = strongly disagree to 5 = strongly agree)  Separately used |
| Likelihood of participation in healthy eating program (i.e. intervention focus) | Would you be willing to participate and learn more about healthy eating for you and your child through ...?   - An individual program - A group program - A family program - A fathers only program | 5-point Likert Scale (1 = very unlikely to 5 = very likely)  Separately used |
| Usefulness of delivery modes | How useful would the following forms of information on healthy eating be to you and your child?   - An online program - An interactive social network program (e.g. online group forum) - A DVD program (e.g. information DVD) - A written program (e.g. information booklet) - A mobile phone program (e.g. SMS or text message) | 5-point Likert Scale (1 = not at all to 5 = very)  Separately used |
| Preference for the location and timing of a nutrition intervention | When and where would you be willing to attend a healthy eating program for you and your child?   - A program in your community - A program after work hours or on weekends | 5-point Likert Scale (1 = very unlikely at all to 5 = very likely)  Separately used |
